# MCmatlab: an open-source, user-friendly, MATLAB-integrated three-dimensional Monte Carlo light transport solver with heat diffusion and tissue damage (Erratum)

**DOI:** 10.1117/1.JBO.26.1.019804

**Published:** 2021-01-29

**Authors:** Dominik Marti, Rikke N. Aasbjerg, Peter E. Andersen, Anders K. Hansen

**Affiliations:** Technical University of Denmark, Department of Photonics Engineering, Roskilde, Denmark

## Abstract

The erratum corrects an error in the originally provided equation for ΔT.

This article [*J. Biomed. Opt.* **23**(12), 121622 (2018) doi: 10.1117/1.JBO.23.12.121622] was originally published on 15 December 2018 with an error in an equation in Sec. 3.1. The provided equation for ΔT on the bottom of column 1 on page 4 in the original article was incorrect. The right-hand side of that equation should have been divided by dx dy dz. The correct equation for ΔT is thus

$$\Delta T=\frac{\Delta t}{c_{V}}(P\mu_{a}F+(T_{x^{-}}-T)\frac{2kk_{x^{-}}}{k+k_{x^{-}}}\frac{1}{dx^{2}}+(T_{x^{+}}-T)\frac{2kk_{x^{+}}}{k+k_{x^{+}}}\frac{1}{dx^{2}}+(T_{y^{-}}-T)\frac{2kk_{y^{-}}}{k+k_{y^{-}}}\frac{1}{dy^{2}}+(T_{y^{+}}-T)\frac{2kk_{y^{+}}}{k+k_{y^{+}}}\frac{1}{dy^{2}}+(T_{z^{-}}-T)\frac{2kk_{z^{-}}}{k+k_{z^{-}}}\frac{1}{dz^{2}}+(T_{z^{+}}-T)\frac{2kk_{z^{+}}}{k+k_{z^{+}}}\frac{1}{dz^{2}})$$

The implementation in the provided MCmatlab was correct at all times, so calculations done with the tool did not suffer from the misprint in the article. The authors wish to credit Dr. Loris Fichera for alerting them to the mistake.

The error was corrected on 19 January 2021.

